# Peri-Operative Risk Factors Associated with Post-Operative Cognitive Dysfunction (POCD): An Umbrella Review of Meta-Analyses of Observational Studies

**DOI:** 10.3390/jcm12041610

**Published:** 2023-02-17

**Authors:** Nikolaj Travica, Mojtaba Lotfaliany, Andrew Marriott, Seyed A. Safavynia, Melissa M. Lane, Laura Gray, Nicola Veronese, Michael Berk, David Skvarc, Hajara Aslam, Elizabeth Gamage, Melissa Formica, Katie Bishop, Wolfgang Marx

**Affiliations:** 1 IMPACT—The Institute for Mental and Physical Health and Clinical Translation, Food & Mood Centre, School of Medicine, Barwon Health, Deakin University, Geelong, VIC 3220, Australia; 2 Barwon Health, Department of Anaesthesia and Pain, University Hospital Geelong, Geelong, VIC 3220, Australia; 3 Department of Anesthesiology, Weill Cornell Medical College, New York, NY 10065, USA; 4 Faculty of Health, School of Medicine, Deakin University, Waurn Ponds, VIC 3216, Australia; 5 Geriatrics Section, Department of Internal Medicine, University of Palermo, 90133 Palermo, Italy; 6 Faculty of Health, School of Psychology, Deakin University, Geelong, VIC 3220, Australia

**Keywords:** surgery, psychiatry, cognition, risk factors, cognitive dysfunction, post-operative, meta-review, neuroscience, peri-operative neurocognitive disorder

## Abstract

This umbrella review aimed to systematically identify the peri-operative risk factors associated with post-operative cognitive dysfunction (POCD) using meta-analyses of observational studies. To date, no review has synthesised nor assessed the strength of the available evidence examining risk factors for POCD. Database searches from journal inception to December 2022 consisted of systematic reviews with meta-analyses that included observational studies examining pre-, intra- and post-operative risk factors for POCD. A total of 330 papers were initially screened. Eleven meta-analyses were included in this umbrella review, which consisted of 73 risk factors in a total population of 67,622 participants. Most pertained to pre-operative risk factors (74%) that were predominantly examined using prospective designs and in cardiac-related surgeries (71%). Overall, 31 of the 73 factors (42%) were associated with a higher risk of POCD. However, there was no convincing (class I) or highly suggestive (class II) evidence for associations between risk factors and POCD, and suggestive evidence (class III) was limited to two risk factors (pre-operative age and pre-operative diabetes). Given that the overall strength of the evidence is limited, further large-scale studies that examine risk factors across various surgery types are recommended.

## 1. Introduction

Peri-operative neurocognitive disorders (PND) describe the cognitive impairments associated with surgery and anesthesia [1,2]. Traditionally described as post-operative delirium (POD) and/or post-operative cognitive dysfunction (POCD), the new PND nomenclature seeks to align these potentially long-term cognitive impairments following surgery and anaesthesia with the clinical diagnostic criteria for neurocognitive disorders applied in the Diagnostic and Statistical Manual for Mental Disorders, fifth edition (DSM-5) [3]. Although the nomenclature is moving towards PND, a large body of evidence has focused on POCD specifically [1,4], and most studies currently refer to these symptoms as POCD (as is this case in this review) [5,6].

POCD is defined as the onset of emergent cognitive impairment in the post-surgical period that exceeds the expected length of time needed to recover from the acute effects of surgery and anaesthesia [7]. The estimated incidence of POCD is 41–75% at one-week post-surgery and 18–45% at three months post-surgery [8] following non-cardiac surgery [9], with higher rates demonstrated post cardiac surgery [10,11]. Longitudinal studies have shown that POCD symptomatology may be prolonged and subtle, affecting several cognitive domains [12]. Deficits in memory and executive function are most commonly reported [7,12]. Although overt symptoms are rarely associated with POCD, some symptoms may be present immediately after surgery and may persist for years post-operatively [13]. POCD may be associated with increased post-operative complications, prolonged hospital stay, earlier retirement, increased utilisation of social and financial assistance, and subsequent higher mortality [4,7].

The aetiology of POCD is underexplored [14], with a multifactorial pathogenesis proposed [7]. Findings from animal experiments and observational human studies suggest neuroinflammation plays a key role, potentially influencing a host of neuropathologies including impaired blood–brain barrier integrity [15], mitochondrial dysfunction, oxidative stress and neuronal apoptosis [16]. Impairments in post-operative endocrinological function characterised by elevated cortisol and hyperglycaemia, in addition to alterations in peripheral circulation and common post-operative complications such as sleep disturbances, pain and prescribed medications have also been purported to contribute to POCD [17,18,19]. In order to establish whether the onset of POCD is associated with the mentioned biological factors, both pre- and post-operative assessments are warranted. This will determine whether any peri-operative changes in these factors relate to potential cognitive changes.

Efficacious treatments and preventative strategies are lacking, likely reflecting a poor understanding of the associated peri-operative risk factors and operative pathways. Preliminary studies have proposed alterations to operative and anaesthetic techniques [7,20], while a number of ongoing studies have attempted to examine the effectiveness of anti-inflammatory, anti-oxidative and pro-neuronal interventions [19].

Recognising the potential risk factors involved in POCD may assist in identifying at-risk populations, which may guide future research and the development of preventative strategies or treatments. At present, a comprehensive body of work that may allow for risk stratification of those most at risk of developing POCD is lacking [21]. One recent umbrella review focused on the risk factors associated with post-operative delirium [22], a common post-operative neurological disorder distinct from POCD and characterised by an acute confused or psychotic state [23]. While there are now several meta-analyses that have synthesised risk factors associated with POCD, no umbrella review has been conducted to assess overall direction or the strength of these associations. Moreover, such an umbrella review allows the findings of previous meta-analyses to be comprehensively compared and contrasted [24]. Therefore, this umbrella review aimed to aggregate and summarise the strength of evidence assessing the association between peri-operative risk factors and POCD derived from numerous meta-analyses of observational studies.

## 2. Materials and Methods

This study was reported in line with the Preferred Reporting Items for Systematic Reviews and Meta-Analyses (PRISMA) guidelines [25] and was prospectively registered in an international registry of systematic reviews (PROSPERO registration no. CRD42021257279).

### 2.1. Literature Search and Selection Criteria

All systematic reviews followed by meta-analyses that examined the potential risk factors associated with POCD using observational study designs (e.g., prospective, case-control) were eligible for inclusion. Risk factors relating to any peri-operative time point (pre-, intra- or post-operative) were included. There was no restriction on the type of surgery, population or age group.

Two independent authors (NT and KB) searched MEDLINE (via PubMed) as well as PsycINFO, EMBASE and the Cochrane databases (via Ovid) from journal inception to December 2022. Key search terms related to the post-operative cognitive outcome (“Post-operative cognitive Dysfunction” OR “Post-operative Cognitive Impairment” OR “POCD” OR “Perioperative Neurocognitive Disorder” OR “Postoperative Neurocognitive Disorder” OR “Delayed Neurocognitive Recovery)” and the meta-analysis study design (“meta-analy” OR “metaanaly” OR “meta reg” OR “metareg”). Retrieved articles were independently screened in duplicate (EG and KB) to identify studies that potentially met the inclusion criteria. Any disagreement between authors over the eligibility of studies was resolved through discussion with a third reviewer (NT).

In line with methods used in prior umbrella reviews [26,27], if two or more meta-analyses were available for the same risk factor, the most recently updated and/or largest meta-analysis was included. A number of risk factors related to specific surgery types where meta-analyses were strictly based on one type of surgery (i.e., coronary artery bypass, carotid endarterectomy). One article categorised meta-analyses by the duration of observed POCD (i.e., acute, midterm, long-term) [28]. In this case, only the meta-analysis relating to the acute phase (POCD within one month of surgery) was used, due to this meta-analysis consisting of the highest number of studies and POCD being most prominent during the first post-operative month [29].

### 2.2. Data Extraction

Duplicate extraction was conducted for the assessment of study quality and evidence synthesis. Extracted data related to study design, sample size, exposures, outcomes and effect estimates. Study-specific risk estimates were extracted (i.e., mean differences, and odds ratios with 95% confidence intervals [95% CIs] for continuous and binary outcomes, respectively). We screened the component studies to ensure that none of the eligible meta-analyses included studies with shared populations. Where required, the study author of the original paper was contacted for further information on relevant data that were not reported.

### 2.3. Data Analysis

The characteristics of the included meta-analyses were summarised by median number of risk factors, total number of participants, median number of cases and their design. We fitted random effect meta-analysis models to pool the extracted effect sizes for each risk factor outcome (odds ratios and standardised mean differences, with 95% CIs). Statistical heterogeneity between studies was evaluated using the I^2^ statistic with a value ≥50% indicative of high heterogeneity and values ≥75% suggestive of very high heterogeneity [30]. We assessed whether there was evidence for small-study effects (i.e., whether smaller studies tend to give substantially larger estimates of effect size compared with larger studies) with the Egger’s regression asymmetry test. A *p* < 0.10 was considered as evidence for small-study effects.

We conducted a test for excess significance for all outcomes [31], which evaluates whether the number of studies with nominally significant results (i.e., *p* < 0.05) within an included meta-analysis, exceeds what would be expected based on the statistical power of the meta-analysis. Excess statistical significance was claimed at two-sided *p* < 0.10.

There is some evidence to show different incidence rates of POCD and severity of symptoms following cardiac related surgery compared to non-cardiac surgery [10,11,32]. As such, for meta-analyses that included studies based on a range of surgery types, additional sensitivity analyses were performed to group studies into cardiac or non-cardiac surgery types. This was carried out to help determine whether the specific risk factor may be dependent on the type of surgery. Data analyses were conducted using R statistical software (version 4.1.3).

### 2.4. Quality Assessment of the Meta-Analyses and Evidence Grading

The quality of all eligible meta-analyses was assessed using the AMSTAR 2 (A Measurement Tool to Assess Systematic Reviews) quality assessment tool [33]. This tool provides a broad assessment of quality, including the identification of flaws that may be associated with the poor conduct of a review.

In line with previous umbrella reviews [26,27], the results of this umbrella review were classified as convincing, highly suggestive, suggestive, weak or no evidence, as defined using the following criteria:

- Convincing (class I); where the number of cases is >1000, statistically significant using a *p* value of <1 × 10^−6^, I^2^ < 50%, 95% prediction interval excludes the null, the largest included individual study has a statistically significant effect (*p* ≤ 0.05), no small-study effects and no excess significance bias.
- Highly suggestive (class II); where the number of cases is >1000, statistically significant using a *p* value of <1 × 10^−6^, the largest included individual study has a statistically significant effect (*p* ≤ 0.05) and class I criteria not met.
- Suggestive (class III); where the number of cases is >1000, *p* value of <1 × 10^−3^ and Class I—II criteria not met.
- Weak (class IV); statistically significant (*p* ≤ 0.05) and class I—III criteria not met.
- No evidence (class V); no statistical significance (*p* > 0.05).

## 3. Results

**Figure 1:**
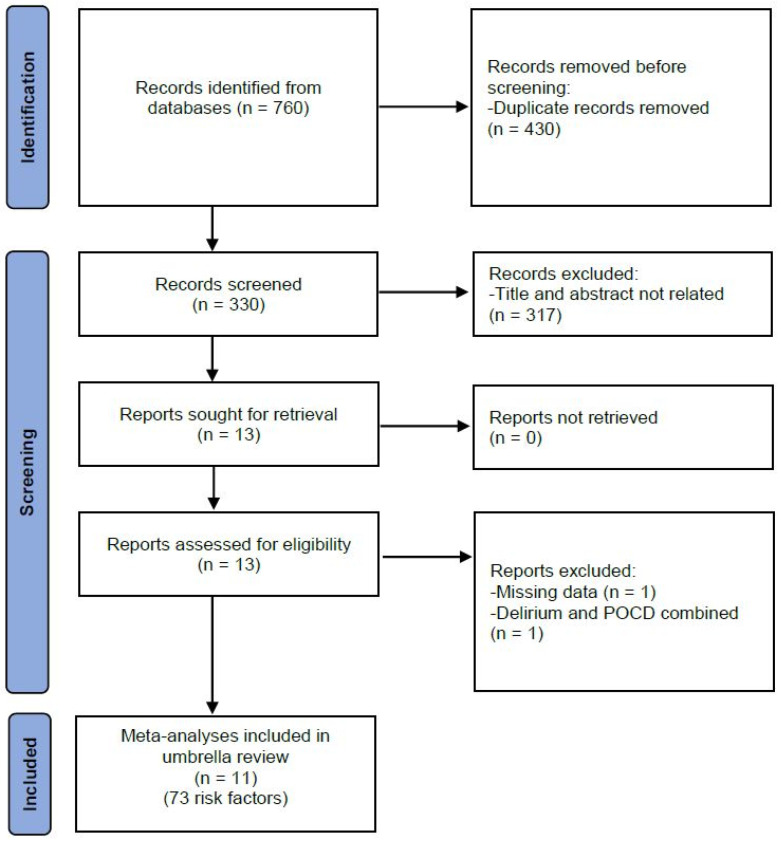
PRISMA flow chart of study selection. Legend: POCD = post-operative cognitive dysfunction.

The systematic search identified 330 de-duplicated articles (Figure 1). After applying the inclusion criteria, 11 reviews of 73 risk factors were included for review [28,34,35,36,37,38,39,40,41,42,43]. A number of the reviews examined the same risk factor but for a different type of surgery. For example, one review examined age as a risk factor following coronary artery bypass, while another examined age following carotid endarterectomy. Consequently, 54 distinct risk factors were considered for review.

### 3.1. Study Characteristics

Most of the meta-analyses were published within the last five years (8/11). The median number of studies included in the meta-analyses for each risk factor was five (range: 2–18), the total number of participants was 67,622, the median number of participants was 727 (range: 74–4247) and the median number of cases (i.e., with the outcome of interest) was 214 (range: 9–1397). Most meta-analyses were based on prospective cohort designs, while two were a combination of case–control and prospective designs [37,42]. As displayed in Table 1, included risk factors primarily focused on pre-operative risk factors (*n* = 54). In contrast, 10 risk factors focused on intraoperative factors and 9 focused on post-operative risk factors. The risk factor exposure variable was either dichotomised (presence versus no presence of the risk factor) or continuous. Most risk factors were coded as categorical variables (*n* = 41), with the remaining 32 risk factors coded as continuous.

### 3.2. Study Results

Overall, 31 of the 73 (42%) risk factors were associated with POCD using a random-effects model (*p* < 0.05) (Table 2), with the following four risk factors surviving a more stringent *p* value (*p* < 1 × 10^−6^) and presenting 95% prediction intervals excluding the null value: Euroscore (coronary artery bypass), which predicts risk of in-hospital mortality after cardiac surgery [28], hypertension (coronary artery bypass) [28], coronary artery bypass time (coronary artery bypass) [28] and hyperperfusion (carotid endarterectomy) [34].

For 19 (26%) risk factors, the largest included study reported a significant association with increased POCD (*p* < 0.05) (Table S1). There was evidence of a small-study effect across four (6%) of the risk factors (Table S1). Heterogeneity was generally low, with most risk factors (51 of 73; 70%) displaying an I^2^ value < 50%. Four risk factors, which included Euroscore (coronary artery bypass), hypertension (coronary artery bypass) [28], coronary artery bypass time (coronary artery bypass) [28] and hyperperfusion (carotid endarterectomy) [34] presented 95% prediction intervals excluding the null value. Evidence of excess significance was present for kidney injury (coronary artery bypass) [28], obesity (cardiac surgery) [35], peripheral vascular disease (coronary artery bypass) [28] and delirium (coronary artery bypass) [28].

### 3.3. Credibility Assessment

When the credibility assessment criteria were applied, no studies presented convincing (class I) or highly suggestive (class II) evidence. Suggestive evidence (class III) was found for associations between two pre-operative risk factors and POCD (age (coronary artery bypass) [28], diabetes (coronary artery bypass) [28]). Weak evidence (class IV) was demonstrated for associations between 31 risk factors and POCD and no significant evidence was shown for associations of a further 40 risk factors with POCD (Table 1 and Table S1). The number of cases was <1000 for a majority of risk factors, contributing to the weak evidence.

### 3.4. Sensitivity Analysis

A number (7/11) of reviews comprised of studies that were conducted on a variety of surgery types. Eight risk factors (1 kg higher body weight, diabetes, education, hypertension, hypercholesterolemia, pre-operative C-reactive protein (CRP), pre-operative interleukin 6, pre-operative S100b) were detected in a mix of surgery types. Therefore, a post-hoc sensitivity analysis divided them into cardiac vs. non-cardiac surgery types. Results indicated that lower education level (*p* = 0.05) and larger pre-operative CRP (*p* < 0.01) were associated with significantly greater risk factors in cardiac surgery than in non-cardiac surgery.

### 3.5. Quality Assessment

The overall quality of included studies was moderate (median score: 17 of 25 using the AMSTAR tool), with limited reporting on several quality assessment items, including sources of funding and assessment of the impact of bias on study results (Table S2).

## 4. Discussion

This is the first umbrella review to provide a comprehensive overview of the observational data assessing the risk factors associated with post-operative cognitive dysfunction (POCD). The strongest evidence supports class III (“suggestive”) associations between pre-operative age and pre-operative diabetes (both in coronary artery bypass surgery) and increased risk of POCD. This umbrella review comprised 73 risk factors in a total population of 67,622 participants. Overall, 31 of the 73 (42%) risk factors were associated with a significantly higher risk of POCD. Most of the meta-analyses comprised of less than 1000 participants.

Despite statistically significant associations between 31 risk factors and POCD, support for most was weak and the credibility was considered class IV or lower. Our findings are unlikely to be attributed to study heterogeneity or small-study effects, given the small portion of meta-analyses (risk factors) displaying significant heterogeneity (30%) and small-study effects (6%). The most plausible explanations for the low level of evidence are likely due to the small sample sizes and the limited number of studies included in the meta-analyses. For example, 28 risk factor meta-analyses (38%) comprised two to three studies, and over two-thirds of included studies had fewer than 1,000 participants. These factors may have limited the power needed to detect statistically significant associations and, in some circumstances, limited formal analysis of excess significance. Smaller samples and meta-analyses may also explain why specific risk factors (i.e., pre-operative diabetes) were significantly associated with POCD in the context of certain surgery types (i.e., coronary artery bypass) although not others (i.e., carotid endarterectomy). These factors could also account for discrepancies in the level of evidence between studies for the same risk factor (i.e., age for coronary artery bypass, class III versus age for carotid endarterectomy, class IV). Another contributing factor is that most associations (*n* = 69/73, 95%) displayed a 95% prediction interval that included the null.

Various explanations may account for the suggestive level of evidence for direct associations between age and POCD in coronary artery bypass surgery. Individuals aged 65 years or more have the greatest risk of developing POCD and experiencing the most severe and persistent symptoms following cardiac surgery [44]. POCD could be influenced by older age increasing the risk of post-coronary artery bypass complications (e.g., delirium, atrial fibrillation, dialysis, reintubation, stroke) that may influence cognitive decline [45,46]. These complications may contribute to exacerbating a number of mechanisms that have been implicated in POCD pathology, such as elevated oxidative stress, neuroinflammation and impaired peripheral and neurocirculation [17]. These complications are likely to increase the length of stay in post-operative intensive care units, which may prolong cognitive recovery [28]. Moreover, older patients admitted for coronary artery bypass surgery are more likely to display a range of pre-existing lifestyle (i.e., smoking) or medical risk factors or comorbidities, such as pain or pre-existing cognitive impairment that may be exacerbated in response to surgery and influence POCD. Together, these risk factors have been conceptualised and grouped as indicators of neurocognitive frailty [47,48], representing the inability to withstand a physiologic stressor such as surgery [49]. Similarly, the proinflammatory processes initiated through the peri-operative period may play a major role in developing neurocognitive disorders amongst older cohorts [50]. Older patients may have a reduced resilience to recover from conditions via higher rates of post-operative neuroinflammation in comparison to younger people [50].

Multiple factors may be involved in the associations between pre-operative diabetes mellitus and POCD in coronary artery bypass surgery. This evidence extends the relatively well-established role of hyperglycaemia in age-related cognitive impairment [51]. Surgery may exacerbate pre-existing neuropathologies, such as neurodegeneration and atherogenesis [52], that are present in diabetes mellitus. The association between POCD and diabetes mellitus in coronary artery bypass patients may reflect the neurological impact of diabetes amongst these patients [53]. Among patients with diabetes mellitus, a meta-analysis that we included reported that POCD risk may further increase with poorer glycaemic control as indexed by higher HbA1C [36]. Given that the surgical stress response may contribute to severe endocrinal disruption characterised by elevated cortisol and blood sugar levels as well as insulin resistance [54,55], this may place individuals with pre-operative diabetes mellitus at an even greater risk of POCD. Diabetes mellitus may also be a marker of shared risk factors for both metabolic syndrome and cognitive decline, such as poor diet, smoking and physical inactivity.

A number of limitations need to be considered when interpreting the findings from the present review. As this review included only outcomes with available meta-analyses, additional risk factors where meta-analyses are currently unavailable were not explored. For instance, a secondary surgical procedure [56] or additional biomarkers such as cortisol and brain-derived neurotrophic factor may be risk factors for POCD [57,58]; however, these have not been included in any previous meta-analyses.

Most of the included meta-analyses examined risk factors that were unadjusted for covariates (univariate data), restricting investigation of independent risk factors. As such, it is difficult to determine whether a certain risk factor is associated with other risk factors and whether they may interact synergistically or confound others. For instance, the mean age of participants amongst most studies was over 60 years, making it difficult to ascertain whether age may have influenced the effects of certain risk factors. Moreover, there is strong and consistent evidence that preoperative cognitive impairment is a strong predictor for POCD [59,60], yet this does not appear to be considered in the meta-analyses.

Although between-study heterogeneity was relatively low, the lack of consensus in the way POCD is defined and measured limits the generalizability of such studies and is a primary driver of the poor class of evidence. In the included reviews, POCD has been evaluated via a wide range of tests, screening tools, and methods of classification. This heterogeneity is an ongoing issue in the field, which has prompted more recent nomenclature recommendations relating to peri-operative neurocognitive disorders [61,62]. In this new classification, POCD is divided into delayed neurocognitive recovery (dNCR) occurring after hospital discharge but < 30 days following surgery, mild/major neurocognitive disorder (NCD), post-operative type occurring ≥30 days and <one year following surgery, and mild/major NCD occurring ≥ one year following surgery [2]. Though classified as distinct entities with potentially different risk factors and therapies, few studies investigated mid- and long-term cognitive decline potentially preventing the identification of important risk factors during these time periods, representing further barriers to conduct subgroup analyses.

Moreover, there were clear between-study differences in the types of surgeries. In addition to a majority of included meta-analyses involving cardiac surgeries, several identified risk factors were solely based on cardiovascular surgeries (51/73). In the few meta-analyses that were not restricted to surgery type, sensitivity analyses demonstrated that greater pre-operative CRP and lower education levels were greater risk factors for POCD following cardiac surgery than other surgery types. This suggests that surgery type is an important consideration when exploring risk factors associated with POCD.

Despite these limitations, this review provides a comprehensive examination of the existing evidence exploring peri-operative risk factors for POCD. Importantly, the review has identified several limitations in the literature in a rapidly evolving area of research. Based on the available data, there are several unexplored POCD risk factors, such as cortisol requiring clarification. Establishing these risk factors should help guide future research to propose factors for prognostic modelling as well as potentially providing hypotheses for intervention studies.

## 5. Conclusions

Notwithstanding the predominantly low level of evidence in the existing literature suggests that a host of risk factors may be associated with POCD. In patients undergoing coronary artery bypass surgery, a suggestive level of evidence was found for independent associations with POCD of pre-operative diabetes mellitus and age. Higher quality evidence that stems from larger prospective studies and a range of surgery types is required to better determine which risk factors are associated with POCD. This knowledge will provide important insight to the identification of at risk populations and potentially guide rigorous prognostic modelling and preventive and treatment studies.

## Figures and Tables

**Table 1:** Summary of included risk factors and their associations with post-operative cognitive dysfunction.

Outcome	Study Design	Level of Comparison	n_Study	Participants, n	n_Cases	Type of Effect Size Metric	Effect Size (95% CI)	95% CI Prediction Intervals	*p* Value	I^2^	Largest Study Effect Size (95% CI)	PUBLICATION Bias	Small-Study Effect or Excess Significance Bias	Evidence Class
**Pre-operative factors**														
AF (transcatheter aortic valve Implantation) [41]	Prospective	Yes vs. no	3	410	58	Odds ratio	0.25 (−0.1, 0.59)	−1.98, 2.47	1.57 × 10^−1^	0%	0.72 (−0.25, 1.68)	No	Neither	**NS**
Age (transcatheter aortic valve implantation) [41]	Prospective	Continuous	2	340	35	Mean difference	0.07 (−0.07, 0.2)	NE	3.34 × 10^−1^	31%	0.15 (−0.03, 0.34)	NE	Neither	**NS**
Age (coronary artery bypass) [28]	Prospective	Continuous	22	2881	1397	Mean difference	0.27 (0.14, 0.41)	−0.38, 0.93	9.47 × 10^−5^	92%	1.49 (1.3, 1.68)	No	Neither	**III**
Age (carotid endarterectomy) [34]	Prospective	Continuous	10	884	122	Mean difference	0.1 (0.03, 0.17)	0.02, 0.18	3.19 × 10^−3^	0%	0.24 (0, 0.49)	No	Neither	**IV**
APOE4 (cardiac) [37]	Prospective	Yes vs. no	3	247	54	Odds ratio	0.26 (−0.13, 0.65)	−2.27, 2.79	1.85 × 10^−1^	0%	0.38 (−0.21, 0.98)	No	Neither	**NS**
APOE4 (non-cardiac) [37]	Prospective	Yes vs. no	3	2928	829	Odds ratio	0.36 (−0.03, 0.75)	−3.89, 4.61	7.16 × 10^−2^	77%	0.68 (−0.92, 2.28)	No	Neither	**NS**
Arrhythmia (coronary artery bypass) [28]	Prospective	Yes vs. no	7	945	400	Odds ratio	0.12 (−0.15, 0.4)	−0.23, 0.48	3.75 × 10^−1^	0%	1.32 (0.19, 2.45)	No	Neither	**NS**
BMI (coronary artery bypass) [28]	Prospective	Continuous	5	910	471	Mean difference	0.14 (−0.01, 0.28)	−0.36, 0.63	6.36 × 10^−2^	75%	0.32 (0.23, 0.42)	No	Neither	**NS**
BMI (transcatheter aortic valve implantation) [41]	Prospective	Continuous	2	340	35	Mean difference	0.04 (−0.06, 0.15)	NE	4.22 × 10^−1^	0%	0.07 (−0.12, 0.26)	NE	No excess significance	**NS**
Cognition: all tests (coronary artery bypass) [28]	Prospective	Continuous	3	155	82	Mean difference	0.2 (0.04, 0.36)	−0.82, 1.22	1.26 × 10^−2^	0%	0.3 (0.05, 0.56)	No	Neither	**IV**
Cognition: MMSE (coronary artery bypass) [28]	Prospective	Continuous	2	120	68	Mean difference	0.23 (0.05, 0.41)	NE	1.19 × 10^−2^	0%	0.31 (0.06, 0.56)	NE	No excess significance	**IV**
Cognitive impairment (coronary artery bypass) [28]	Prospective	Yes vs. no	4	749	214	Odds ratio	0.06 (−0.19, 0.3)	−0.48, 0.6	6.50 × 10^−1^	0%	0.25 (−0.21, 0.71)	No	Neither	**NS**
Cognitive impairment (transcatheter aortic valve implantation) [41]	Prospective	Yes vs. no	2	340	35	Odds ratio	−0.13 (−0.73, 0.47)	NE	6.72 × 10^−1^	8%	−0.01 (−0.54, 0.52)	NE	No excess significance *	**NS**
Contralateral stenonsis (carotid endarterectomy) [34]	Prospective	Yes vs. no	3	252	92	Odds ratio	0.32 (−0.18, 0.83)	−2.96, 3.61	2.12 × 10^−1^	0%	0.35 (−0.86, 1.56)	No	Neither	**NS**
C-reactive protein [42]	Prospective	Continuous	10	594	211	Mean difference	0.21 (0.08, 0.33)	−0.16, 0.58	1.14 × 10^−3^	56%	0.64 (0.33, 0.94)	No	Neither	**NS**
C-reactive protein (hip arthroplasty) [43]	Prospective	Continuous	8	744	208	Mean difference	0.23 (0.11, 0.35)	−0.11, 0.56	2.01 × 10^−4^	55%	0.5 (0.27, 0.73)	No	Either	**IV**
Depression (coronary artery bypass) [28]	Prospective	Yes vs. no	2	330	188	Odds ratio	0.68 (0.06, 1.3)	NE	3.12 × 10^−2^	62%	1.01 (0.43, 1.58)	NE	Neither	**IV**
Depression: all test (coronary artery bypass) [28]	Prospective	Yes vs. no	4	426	127	Mean difference	0.28 (−0.1, 0.67)	−1.53, 2.1	1.48 × 10^−1^	92%	0.82 (0.61, 1.02)	No	Neither	**NS**
Diabetes [36]	Prospective	Yes vs. no	13	2554	815	Odds ratio	0.16 (0.01, 0.32)	−0.33, 0.65	4.13 × 10^−2^	71%	2.17 (0.36, 3.98)	No	No small-study effect *	**IV**
Diabetes (transcatheter aortic valve implantation) [41]	Prospective	Yes vs. no	2	340	35	Odds ratio	−0.15 (−0.64, 0.33)	NE	5.34 × 10^−1^	0%	−0.09 (−0.62, 0.44)	NE	No excess significance *	**NS**
Diabetes (carotid endarterectomy) [34]	Prospective	Yes vs. no	12	1888	493	Odds ratio	0.12 (−0.06, 0.3)	−0.22, 0.46	1.83 × 10^−1^	15%	0.9 (0.34, 1.46)	No	Neither	**NS**
Diabetes (coronary artery bypass) [28]	Prospective	Yes vs. no	17	2968	1273	Odds ratio	0.2 (0.1, 0.3)	0.06, 0.34	4.63 × 10^−5^	5%	0.57 (0.08, 1.06)	No	Neither	**III**
Dyslipidemia (carotid endarterectomy) [34]	Prospective	Yes vs. no	8	766	374	Odds ratio	0.03 (−0.22, 0.28)	−0.29, 0.34	8.38 × 10^−1^	0%	0.53 (−0.66, 1.72)	No	Neither	**NS**
Dyslipidemia (coronary artery bypass) [28]	Prospective	Yes vs. no	6	840	227	Odds ratio	0.08 (−0.17, 0.33)	−0.54, 0.71	5.13 × 10^−1^	36%	0.86 (0.24, 1.47)	No	Neither	**NS**
Education [38]	Prospective	Continuous	8	2535	680	Odds ratio	−0.06 (−0.09, −0.03)	−0.14, 0.01	3.74 × 10^−5^	44%	−0.01 (−0.06, 0.03)	Yes	Small-study effects	**IV**
Education (coronary artery bypass) [28]	Prospective	Continuous	6	538	243	Mean difference	0.14 (0.05, 0.22)	0.02, 0.26	1.67 × 10^−3^	0%	0.3 (−0.04, 0.63)	No	Neither	**IV**
Euroscore (coronary artery bypass) [28]	Prospective	Continuous	4	582	233	Mean difference	0.23 (0.14, 0.31)	0, 0.45	2.09 × 10^−7^	10%	0.3 (0.16, 0.45)	No	Neither	**IV**
Hypercholestrolemia [40]	Prospective	Yes vs. no	12	1538	436	Odds ratio	−0.04 (−0.12, 0.04)	−0.13, 0.05	3.47 × 10^−1^	0%	0.86 (0.24, 1.47)	No	Neither	**NS**
Hypertension [39]	Prospective	Yes vs. no	24	4247	1385	Odds ratio	0.03 (−0.03, 0.1)	−0.15, 0.22	3.28 × 10^−1^	35%	0.92 (0.02, 1.83)	No	No small-study effect *	**NS**
Hypertension (carotid endarterectomy) [34]	Prospective	Yes vs. no	12	1887	1266	Odds ratio	−0.01 (−0.15, 0.13)	−0.17, 0.15	9.00 × 10^−1^	0%	0.61(−1.01, 2.23)	No	Neither	**NS**
Hypertension (transcatheter aortic valve implantation) [41]	Prospective	Yes vs. no	2	340	35	Odds ratio	0.15 (−1.01, 1.32)	NE	7.99 × 10^−1^	28%	0.59 (−0.54, 1.72)	NE	No excess significance *	**NS**
Hypertension (coronary artery bypass) [28]	Prospective	Yes vs. no	15	2115	871	Odds ratio	0.36 (0.21, 0.51)	−0.03, 0.75	4.91 × 10^−6^	34%	1 (0.6, 1.41)	No	Neither	**IV**
Interleukin 1β (hip arthroplasty) [43]	Prospective	Continuous	5	247	90	Mean difference	0.19 (0.04, 0.34)	−0.17, 0.55	1.14 × 10^−2^	26%	0.39 (0.09, 0.68)	No	Neither	**IV**
Interleukin 6 [42]	Prospective	Continuous	16	986	373	Mean Difference	0.15 (0.08, 0.22)	0, 0.29	2.43 × 10^−5^	17%	0.6 (0.34, 0.86)	No	Neither	**IV**
Interleukin 6 (hip arthroplasty) [43]	Prospective	Continuous	6	699	182	Mean difference	0.1 (0.03, 0.17)	0, 0.21	7.78 × 10^−3^	0%	0.24(−0.08, 0.57)	No	Neither	**IV**
Kidney injury (coronary artery bypass) [28]	Prospective	Yes vs. no	4	749	275	Odds ratio	0.11 (−0.51, 0.72)	−2.26, 2.47	7.35 × 10^−1^	54%	0.79 (0.01, 1.57)	No	Excess significance bias	**NS**
LVEF% (coronary artery bypass) [28]	Prospective	Continuous	9	1225	623	Mean Difference	0.14 (0.04, 0.24)	−0.15, 0.43	4.72 × 10^−3^	62%	0.64 (0.32, 0.97)	Yes	Small-study effects	**IV**
Male sex (transcatheter aortic valve implantation) [41]	Prospective	Male vs. female	2	340	35	Odds ratio	0.13 (−0.26, 0.53)	NE	5.12 × 10^−1^	0%	0.27 (−0.7, 1.23)	NE	No excess significance *	**NS**
Male sex (carotid endarterectomy) [34]	Prospective	Male vs. female	12	1888	1397	Odds ratio	−0.05 (−0.15, 0.05)	−0.16, 0.06	3.28 × 10^−1^	0%	0.88 (−0.32, 2.07)	No	Neither	**NS**
Male sex (coronary artery bypass) [28]	Prospective	Male vs. female	18	2403	1069	Odds ratio	0.01 (−0.11, 0.14)	−0.12, 0.15	8.25 × 10^−1^	0%	0.65 (−1.12, 2.43)	No	Neither	**NS**
Obesity [35]	Prospective	Yes vs. no	3	696	164	Odds ratio	0.33 (−0.16, 0.81)	−5.03, 5.68	1.85 × 10^−1^	78%	1.76 (0.19, 3.33)	No	Excess significance	**NS**
One kg higher body weight (cardiac) [35]	Prospective	Yes vs. no	2	293	80	Odds ratio	−0.11 (−0.51, 0.28)	NE	5.67 × 10^−1^	49%	0 (−0.06, 0.06)	NE	No excess significance *	**NS**
Peripheral vascular disease (coronary artery bypass) [28]	Prospective	Yes vs. no	4	856	329	Odds ratio	0.04 (−0.49, 0.57)	−2, 2.09	8.69 × 10^−1^	57%	0.81 (0.09, 1.53)	No	Excess significance bias	**NS**
Pre-operative symptoms (carotid endarterectomy) [34]	Prospective	Yes vs. no	8	1196	596	Odds ratio	0.17 (−0.01, 0.35)	−0.06, 0.39	6.72 × 10^−2^	0%	0.85 (0.02, 1.69)	No	Neither	**IV**
Previous MI <90 days (coronary artery bypass) [28]	Prospective	Yes vs. no	3	418	152	Odds ratio	0.06 (−0.29, 0.42)	−2.26, 2.39	7.24 × 10^−1^	0%	0.27 (−0.35, 0.88)	No	Neither	**NS**
Previous MI history (coronary artery bypass) [28]	Prospective	Yes vs. no	7	1011	455	Odds ratio	0.08 (−0.11, 0.27)	−0.33, 0.49	3.93 × 10^−1^	25%	0.33 (−0.12, 0.79)	No	Neither	**NS**
Previous stroke, TIA, CVA (coronary artery bypass) [28]	Prospective	Yes vs. no	5	745	287	Odds ratio	0.49 (0.21, 0.77)	0.04, 0.94	5.82 × 10^−4^	0%	0.86 (0.35, 1.38)	No	Neither	**IV**
s100b [42]	Prospective, case-control	Continuous	5	232	108	Mean Difference	0.27 (0.02, 0.53)	−0.62, 1.17	3.57 × 10^−2^	74%	1.13 (0.69, 1.56)	No	Neither	**IV**
S100b (hip arthroplasty) [43]	Prospective	Continuous	3	245	58	Mean difference	0.23 (0.1, 0.36)	−0.71, 1.17	5.96 × 10^−4^	5%	0.31 (−0.03, 0.64)	No	Neither	**IV**
Smoking current/history (coronary artery bypass) [28]	Prospective	Yes vs. no	9	1560	713	Odds ratio	0.02 (−0.25, 0.28)	−0.78, 0.82	8.94 × 10^−1^	68%	1.49 (−0.28, 3.27)	No	Neither	**NS**
Statin use [40]	Prospective	Yes vs. no	8	1804	445	Odds ratio	−0.15 (−0.31, 0)	−0.54, 0.24	5.76 × 10^−2^	41%	0.17 (−0.14, 0.48)	No	Neither	**IV**
Statin (carotid endarterectomy) [34]	Prospective	Yes vs. no	3	1279	741	Odds ratio	−0.31 (−0.49, −0.14)	−1.77, 1.14	4.31 × 10^−4^	21%	−0.18 (−0.56, 0.2)	No	Neither	**IV**
Stroke/TIA (transcatheter aortic valve implantation) [41]	Prospective	Yes vs. no	2	340	35	Odds ratio	0.17 (−0.41, 0.74)	NE	5.70 × 10^−1^	0%	0.56 (−0.42, 1.54)	NE	No excess significance *	**NS**
Tumour necrosis factor alpha (hip arthroplasty) [43]	Prospective	Continuous	5	412	127	Mean difference	0.17 (0.08, 0.27)	0.02, 0.33	4.65 × 10^−4^	0%	0.26 (0.03, 0.49)	NE	Neither	**IV**
**Intraoperative**														
Aortic cross-clamping time (coronary artery bypass) [28]	Prospective	Continuous	7	608	275	Mean Difference	0.13 (0.05, 0.21)	0, 0.26	2.46 × 10^−3^	6%	0.52 (0.19, 0.85)	No	Neither	**IV**
CPB time (coronary artery bypass) [28]	Prospective	Continuous	13	1829	942	Mean Difference	0.1 (0.06, 0.15)	0.05, 0.16	8.88 × 10^−8^	0%	0.49 (0.11, 0.86)	Yes	Small-study effects	**IV**
Cross-clamping duration (carotid endarterectomy) [34]	Prospective	Continuous	10	892	130	Mean Difference	0.1 (0.02, 0.19)	−0.09, 0.29	1.38 × 10^−2^	29%	0.48 (0.14, 0.82)	Yes	Small-study effects	**IV**
Cerebral protection device (Transcatheter Aortic Valve Implantation) [41]	Prospective	Yes vs. no	2	127	47	Odds ratio	0 (−0.41, 0.4)	NE	9.91 × 10^−1^	0%	0.18 (−0.37, 0.74)	NE	No excess significance *	**NS**
Hyperperfusion (carotid endarterectomy) [34]	Prospective	Yes vs. no	5	417	52	Odds ratio	1.97 (1.55, 2.39)	1.29, 2.65	4.18 × 10^−20^	0%	2.91 (1.06, 4.75)	No	Neither	**IV**
Intubation time (coronary artery bypass) [28]	Prospective	Continuous	6	1193	589	Mean Difference	0.41 (−0.29, 1.1)	−2.19, 3	2.5 × 10^−1^	99%	1.7 (1.61, 1.8)	No	Neither	**NS**
Number of grafts (coronary artery bypass) [28]	Prospective	Continuous	7	1113	536	Mean Difference	0.07 (0.01, 0.12)	−0.01, 0.14	2.96 × 10^−2^	0%	0.26 (−0.03, 0.54)	Yes	Small-study effects	**IV**
Selective shunting placement (carotid endarterectomy) [34]	Prospective	Yes vs. no	2	220	9	Odds ratio	0.7 (−0.07, 1.46)	NE	7.62 × 10^−2^	0%	0.76 (−0.43, 1.95)	NE	No excess significance *	**NS**
Surgery duration (coronary artery bypass) [28]	Prospective	Continuous	6	727	261	Mean Difference	0.13 (0.06, 0.21)	0.03, 0.24	3.174 × 10^−4^	0%	0.24 (0.05, 0.43)	No	Neither	**IV**
Total microemboli (coronary artery bypass) [28]	Prospective	Continuous	4	791	434	Mean Difference	0.09 (0.02, 0.15)	−0.07, 0.24	1.68 × 10^−2^	0%	0.18 (−0.1, 0.47)	No	Neither	**IV**
**Post-operative**														
Arrhythmia (coronary artery bypass) [28]	Prospective	Yes vs. no	6	1045	487	Odds ratio	0.19 (0.01, 0.36)	−0.07, 0.44	4.22 × 10^−2^	0%	0.65 (−0.02, 1.32)	No	Neither	**IV**
C-reactive protein (hip arthroplasty) [43]	Prospective	Continuous	2	74	25	Mean difference	0.83 (−0.31, 1.96)	NE	1.53 × 10^−1^	96%	1.41 (1.1, 1.72)	No	Neither	**NS**
Delirium (coronary artery bypass) [28]	Prospective	Yes vs. no	3	355	148	Odds ratio	1 (0.46, 1.54)	−2.92, 4.92	2.54 × 10^−4^	6%	2.02 (0.39, 3.65)	No	Excess significance bias	**IV**
LOS in ICU (coronary artery bypass) [28]	Prospective	Continuous	7	1059	547	Mean Difference	0.43 (−0.13, 1)	−1.64, 2.5	1.31 × 10^−1^	99%	1.49 (1.4, 1.58)	No	Neither	**NS**
Interleukin 6 (hip arthroplasty) [43]	Prospective	Continuous	3	131	60	Mean difference	0.52 (0.32, 0.72)	−1.18, 2.22	3.41 × 10^−1^	24%	0.63 (0.31, 0.95)	No	Neither	**NS**
Interleukin 1β (hip arthroplasty) [43]	Prospective	Continuous	3	131	60	Mean difference	0.12 (−0.1, 0.34)	−2.01, 2.25	2.99 × 10^−1^	39%	0.37 (0.04, 0.71)	No	Neither	**NS**
Stroke (transcatheter aortic valve implantation) [41]	Prospective	Yes vs. no	3	325	47	Odds ratio	−0.35 (−1.24, 0.55)	−7.8, 7.1	4.48 × 10^−1^	22%	0.3 (−1.51, 2.1)	No	Neither	**NS**
S100b (hip arthroplasty) [43]	Prospective	Continuous	3	131	60	Mean difference	0.51 (0.3,0.72)	−1.38, 2.39	1.85 × 10^−1^	31%	0.62 (0.3, 0.9)	No	Neither	**NS**
Tumour necrosis factor alpha (hip arthroplasty) [43]	Prospective	Continuous	2	97	42	Mean difference	0.21 (0.01, 0.41)	NE	3.97 × 10^−2^	0%	0.3 (−0.02, 0.62)	NE	No excess significance *	**IV**

Legend: * Either tests for small-study effect, excess significance, or both, could not be conducted due to small sample size of included studies. Evidence class criteria—class I (convincing): statistical significance at *p* < 10^−6^, >1000 cases (or >20,000 participants for continuous outcomes), the largest component study reported a significant effect (*p* < 0.05); the 95% prediction interval excluded the null, no large heterogeneity (I^2^ < 50%), no evidence of small-study effects (*p* > 0.10) and excess significance bias (*p* > 0.10); class II (highly suggestive): significance at *p* < 10^−6^, >1000 cases (or >20,000 participants for continuous outcomes), the largest component study reported a significant effect (*p* ≤ 0.05); class III (suggestive): statistical significance at *p* < 10^−3^, >1000 cases (or >20,000 participants for continuous outcomes); and class IV (weak): the remaining significant associations at *p* < 0.05; AF = atrial fibrillation, APOE4 = Apolipoprotein E4, BMI = body mass index, CPB = cardiopulmonary bypass, CVA = cerebrovascular accident, ICU = intensive care unit, LOS = length of stay, LVEF% = left ventricular ejection fraction, MI = myocardial infarction, MMSE = Mini Mental State Examination, NS = non-significant (*p* >0.05), S100B = S100 calcium-binding protein B, TIA = transient ischemic attack.

**Table 2:** Summary of risk factors displaying significant (*p* ≤ 0.05) associations with post-operative cognitive dysfunction.

Outcome	Evidence Class	Study, n	Participants, n	Effect Size (95% CI)	*p* Value	I^2^
**Pre-operative factors**						
Age (coronary artery bypass)	III	22	2881	0.27 (0.14, 0.41)	9.47 × 10^−5^	92%
Age (carotid endarterectomy)	IV	10	884	0.1 (0.03, 0.17)	3.19 × 10^−3^	0%
Cognition: All tests (coronary artery bypass)	IV	3	155	0.2 (0.04, 0.36)	1.26 × 10^−2^	0%
Cognition: MMSE (coronary artery bypass)	IV	2	120	0.23 (0.05, 0.41)	1.19 × 10^−2^	0%
C-reactive protein (hip arthroplasty)	IV	8	744	0.23 (0.11, 0.35)	2.01 × 10^−4^	55%
Depression (coronary artery bypass)	IV	2	330	0.68 (0.06, 1.3)	3.12 × 10^−2^	62%
Diabetes	IV	13	2554	0.16 (0.01, 0.32)	4.13 × 10^−2^	71%
Diabetes (coronary artery bypass)	III	17	2968	0.2 (0.1, 0.3)	4.63 × 10^−5^	5%
Education	IV	8	2535	−0.06 (−0.09, −0.03)	3.74 × 10^−5^	44%
Education (coronary artery bypass)	IV	6	538	0.14 (0.05, 0.22)	1.67 × 10^−3^	0%
Euroscore (coronary artery bypass)	IV	4	582	0.23 (0.14, 0.31)	2.09 × 10^−7^	10%
Hypertension (coronary artery bypass)	IV	15	2115	0.36 (0.21, 0.51)	4.91 × 10^−6^	34%
Interleukin 1β (hip arthroplasty)	IV	5	247	0.19 (0.04, 0.34)	1.14 × 10^−2^	26%
Interleukin 6	IV	16	986	0.15 (0.08, 0.22)	2.43 × 10^−5^	17%
Interleukin 6 (hip arthroplasty)	IV	6	699	0.1 (0.03, 0.17)	7.78 × 10^−3^	0%
LVEF% (coronary artery bypass)	IV	9	1225	0.14 (0.04, 0.24)	4.72 × 10^−3^	62%
Previous stroke, TIA, CVA (coronary artery bypass)	IV	5	745	0.49 (0.21, 0.77)	5.82 × 10^−4^	0%
S100b	IV	5	232	0.27 (0.02, 0.53)	3.57 × 10^−2^	74%
S100b (hip arthroplasty)	IV	3	245	0.23 (0.1, 0.36)	5.96 × 10^−4^	5%
Statin (carotid endarterectomy)	IV	3	1279	−0.31 (−0.49, −0.14)	4.31 × 10^−4^	21%
Tumour necrosis factor alpha (hip arthroplasty)	IV	5	412	0.17 (0.08, 0.27)	4.65 × 10^−4^	0%
**Intra-operative factors**						
Aortic cross-clamping time (coronary artery bypass)	IV	7	608	0.13 (0.05, 0.21)	2.46 × 10^−3^	6%
CPB time (coronary artery bypass)	IV	13	1829	0.1 (0.06, 0.15)	8.88 × 10^−8^	0%
Cross-clamping duration (carotid endarterectomy)	IV	10	893	0.1 (0.02, 0.19)	1.38 × 10^−2^	29%
Hyperperfusion (carotid endarterectomy)	IV	5	417	1.97 (1.55, 2.39)	4.18 × 10^−20^	0%
Number of grafts (coronary artery bypass)	IV	7	1113	0.07 (0.01, 0.12)	2.96 × 10^−2^	0%
Surgery duration (coronary artery bypass)	IV	6	727	0.13 (0.06, 0.21)	3.17 × 10^−4^	0%
Total microemboli (coronary artery bypass)	IV	4	791	0.09 (0.02, 0.15)	1.68 × 10^−2^	0%
**Post-operative factors**						
Arrhythmia (coronary artery bypass)	IV	6	1045	0.19 (0.01, 0.36)	4.22 × 10^−2^	0%
Delirium (coronary artery bypass)	IV	3	355	1 (0.46, 1.54)	2.54 × 10^−4^	6%
Tumour necrosis factor alpha (hip arthroplasty)	IV	2	97	0.21 (0.01, 0.41)	3.97 × 10^−2^	0%

Abbreviations: CPB = cardiopulmonary bypass, CVA = cerebrovascular accident, LVEF% = left ventricular ejection fraction, MMSE = Mini Mental State Examination, S100b = S100 calcium-binding protein B, TIA = transient ischemic attack.

## Data Availability

Data are contained within the article or Supplementary Materials.

## Supplementary Materials

The following supporting information can be downloaded at: https://www.mdpi.com/article/10.3390/jcm12041610/s1, Table S1: Credibility assessment details; Table S2: AMSTAR ratings.
