# Supplementary material for: Peri-Operative Risk Factors Associated with Post-Operative Cognitive Dysfunction (POCD): An Umbrella Review of Meta-Analyses of Observational Studies
Source: J Clin Med. 2023 Feb 17;12(4):1610. doi: 10.3390/jcm12041610 (PMC9965885; doi:10.3390/jcm12041610)
Supplement: Supplementary file 1 [file jcm-12-01610-s001.zip › Supplementary Table S1 & S2.pdf]

Supplementary Table S1. Credibility assessment details

Outcome	P < 10^{-6}	p<10^{-3}	p<0.05	>1000 cases (or >20,000)	Largest component study reported P < 0.05	95% prediction interval excluded the null	I^2 < 50%	Evidence of small-study effects	Evidence of excess significance bias	Class
**Pre-operative factors**										
Atrial fibrillation (Transcatheter Aortic Valve Implantation)	No	No	No	No	No	No	Yes	No	No	**V**
Age (Transcatheter aortic valve implantation)	No	No	No	No	No	No	Yes	No	No	**V**
Age (Coronary artery bypass)	No	Yes	Yes	Yes	No	No	No	No	No	**III**
Age (carotid endarterectomy)	No	Yes	Yes	No	No	No	Yes	No	No	**IV**
APOE4 (cardiac)	No	No	No	No	Yes	No	Yes	No	No	**V**
APOE4 (non-cardiac)	No	No	No	No	Yes	No	No	No	No	**V**
Arrhythmia (coronary artery bypass)	No	No	No	No	No	No	Yes	No	No	**V**
BMI (coronary artery bypass)	No	No	No	No	No	No	Yes	No	No	**V**

BMI (Transcatheter aortic valve implantation)	No	No	No	No	No	No	Yes	NE	No	**V**
Cognition: All tests (coronary artery bypass)	No	No	Yes	No	No	No	Yes	No	No	**IV**
Cognition: MMSE (coronary artery bypass)	No	No	Yes	No	No	No	Yes	NE	No	**IV**
Cognitive impairment (coronary artery bypass)	No	No	No	No	No	No	Yes	No	No	**V**
Cognitive impairment (Transcatheter Aortic Valve Implantation)	No	No	No	No	No	No	Yes	NE	No	**V**
Contralateral stenonsis (carotid endarterectomy)	No	No	No	No	No	No	Yes	No	No	**V**
C-reactive protein	No	No	No	No	No	No	No	No	No	**V**
C-reactive protein (hip arthroplasty)	No	Yes	Yes	No	No	No	No	No	No	**IV**
Depression (coronary artery bypass)	No	No	Yes	No	Yes	No	No	No	No	**IV**
Depression: All tests (coronary artery bypass)	No	No	No	No	No	No	No	No	No	**V**
Diabetes	No	No	Yes	No	Yes	No	No	No	NE	**IV**

Diabetes (Transcatheter Aortic Valve Implantation)	No	No	No	No	No	No	Yes	NE	No	**V**
Diabetes (carotid endarterectomy)	No	No	No	No	Yes	No	Yes	No	No	**V**
Diabetes (coronary artery bypass)	No	Yes	Yes	Yes	Yes	No	Yes	No	No	**III**
Dyslipidemia (carotid endarterectomy)	No	No	No	No	No	No	Yes	No	No	**V**
Dyslipidemia (coronary artery bypass)	No	No	No	No	No	No	Yes	No	No	**V**
Education	No	Yes	Yes	No	Yes	No	Yes	No	No	**IV**
Education (coronary artery bypass)	No	Yes	Yes	No	Yes	No	Yes	No	No	**IV**
Euroscore (coronary artery bypass)	Yes	Yes	Yes	No	Yes	Yes	Yes	No	No	**IV**
Hypercholestrolemi a	No	No	No	No	No	No	Yes	No	No	**V**
Hypertension	No	No	No	Yes	No	No	Yes	No	NE	**V**
Hypertension (carotid endarterectomy)	No	No	No	Yes	No	No	Yes	No	No	**V**
Hypertension (Transcatheter Aortic Valve Implantation)	No	No	No	No	No	No	Yes	NE	No	**V**

Hypertension (coronary artery bypass)	Yes	Yes	Yes	No	Yes	Yes	Yes	No	No	**IV**
Interleukin 1β (hip arthroplasty)	No	No	Yes	No	No	No	Yes	No	No	**IV**
Interleukin 6	No	Yes	Yes	No	No	No	Yes	No	No	**IV**
Interleukin 6 (hip arthroplasty)	No	Yes	Yes	No	Yes	No	Yes	No	No	**IV**
Kidney injury (coronary artery bypass)	No	No	No	No	No	No	No	No	Yes	**V**
LVEF% (coronary artery bypass)	No	Yes	Yes	No	Yes	No	No	Yes	No	**IV**
Male sex (Transcatheter Aortic Valve Implantation)	No	No	No	No	No	No	Yes	NE	No	**V**
Male sex (carotid endarterectomy)	No	No	No	Yes	No	No	Yes	No	No	**V**
Male sex (coronary artery bypass)	No	No	No	Yes	No	No	Yes	No	No	**V**
Obesity	No	No	No	No	No	No	No	No	Yes	**V**
One kg higher body weight (cardiac)	No	No	No	No	No	No	Yes	NE	No	**V**
Peripheral vascular disease (coronary artery bypass)	No	No	No	No	No	No	No	No	Yes	**V**
Pre-operative symptoms (carotid endarterectomy)	No	No	No	No	Yes	No	Yes	No	No	**IV**

Previous MI <90 days (coronary artery bypass)	No	No	No	No	No	No	Yes	No	No	**V**
Previous MI history (coronary artery bypass)	No	No	No	No	No	No	Yes	No	No	**V**
Previous stroke, TIA, CVA (coronary artery bypass)	No	Yes	Yes	No	Yes	No	Yes	No	No	**IV**
s100b	No	No	Yes	No	Yes	No	No	No	No	**IV**
S100b (hip arthroplasty)	No	Yes	Yes	No	No	No	Yes	No	No	**IV**
Smoking current/history (coronary artery bypass)	No	No	No	No	No	No	No	No	No	**V**
Statin use	No	No	No	No	No	No	Yes	No	No	**IV**
Statin (carotid endarterectomy)	No	Yes	Yes	No	No	No	Yes	No	No	**IV**
Stroke/TIA (Transcatheter Aortic Valve Implantation)	No	No	No	No	No	No	Yes	NE	No	**V**
Tumour Necrosis Factor alpha (hip arthroplasty)	No	Yes	Yes	No	No	No	Yes	No	No	**IV**
**Intraoperative factors**										
Aortic cross clamping time	No	Yes	Yes	No	No	No	Yes	No	No	**IV**

(coronary artery bypass)										
CPB time (coronary artery bypass)	Yes	Yes	Yes	No	No	Yes	Yes	Yes	No	**IV**
Cross-clamping duration (carotid endarterectomy)	No	No	Yes	No	No	No	Yes	Yes	No	**IV**
Cerebral protection device (Transcatheter Aortic Valve Implantation)	No	No	No	No	No	No	Yes	NE	No	**V**
Hyperperfusion (carotid endarterectomy)	Yes	Yes	Yes	No	Yes	Yes	Yes	No	No	**IV**
Intubation time (coronary artery bypass)	No	No	No	No	No	No	No	No	No	**V**
Number of grafts (coronary artery bypass)	No	No	Yes	No	No	No	Yes	Yes	No	**IV**
Selective shunting placement (carotid endarterectomy)	No	No	No	No	No	No	Yes	No	No	**V**
Surgery duration (coronary artery bypass)	No	Yes	Yes	No	No	No	Yes	No	No	**IV**
Total microemboli (coronary artery bypass)	No	No	Yes	No	No	No	Yes	No	No	**IV**
**Post-operative factors**										

Arrhythmia (coronary artery bypass)	No	No	Yes	No	Yes	No	Yes	No	No	**IV**
C-reactive protein (hip arthroplasty)	No	No	No	No	No	No	No	No	No	**V**
Delirium (coronary artery bypass)	No	Yes	Yes	No	Yes	No	Yes	No	Yes	**IV**
LOS in ICU (coronary artery bypass)	No	No	No	No	No	No	No	No	No	**V**
Interleukin 6 (hip arthroplasty)	No	No	No	No	No	No	Yes	No	No	**V**
Interleukin 1β (hip arthroplasty)	No	No	No	No	No	No	Yes	No	No	**V**
Stroke (Transcatheter Aortic Valve Implantation)	No	No	No	No	No	No	Yes	No	No	**V**
S100b (hip arthroplasty)	No	No	No	No	No	No	Yes	No	No	**V**
Tumour Necrosis Factor alpha (hip arthroplasty)	No	No	Yes	No	Yes	No	Yes	NE	No	**IV**

Legend: AF = atrial fibrillation, APOE4 = Apolipoprotein E4, BMI = BMI, CPB = cardiopulmonary bypass, CVA= cerebrovascular accident, ICU = intensive care unit, LOS = length of stay, LVEF% = Left ventricular ejection fraction, MI = myocardial infarction, MMSE =Mini Mental State Examination, S100B = S100 calcium-binding protein B, TIA = Transient ischemic attack

Supplementary Table S2. AMSTAR ratings

	AMSTAR Items*																
**Authors / Reference**	**Year**	**1**	**2**	**3**	**4**	**5**	**6**	**7**	**8**	**9**	**10**	**11**	**12**	**13**	**14**	**15**	**16**	**TOTAL**
Aceto et al.[1]	**2019**	**2**	**1**	**2**	**2**	**2**	**2**	**1**	**2**	**2**	**0**	**2**	**0**	**2**	**1**	**2**	**2**	**25**
Cao et al.[2]	**2014**	**2**	**0**	**2**	**1**	**2**	**2**	**1**	**2**	**0**	**0**	**2**	**0**	**0**	**2**	**2**	**2**	**20**
Feinkohl et al.[3]	**2017**	**2**	**1**	**2**	**1**	**0**	**0**	**1**	**2**	**2**	**0**	**2**	**0**	**0**	**2**	**2**	**2**	**19**
Feinkohl et al.[4]	**2016**	**2**	**0**	**2**	**1**	**0**	**0**	**0**	**2**	**0**	**0**	**2**	**0**	**0**	**2**	**2**	**2**	**15**
Feinkohl et al. (2)[5]	**2017**	**2**	**1**	**2**	**1**	**0**	**0**	**1**	**2**	**2**	**0**	**2**	**0**	**0**	**2**	**2**	**2**	**19**
Feinkohl et al.[6]	**2018**	**2**	**1**	**2**	**1**	**0**	**0**	**1**	**2**	**2**	**0**	**2**	**0**	**0**	**2**	**2**	**2**	**19**
Feinkohl et al. (3)[7]	**2017**	**2**	**0**	**2**	**1**	**0**	**0**	**1**	**1**	**2**	**0**	**1**	**0**	**0**	**2**	**2**	**2**	**16**
Fu et al.[8]	**2021**	**2**	**0**	**2**	**1**	**2**	**2**	**1**	**1**	**0**	**0**	**2**	**0**	**0**	**2**	**2**	**2**	**19**
Ghezzi et al.[9]	**2019**	**2**	**0**	**2**	**1**	**2**	**2**	**1**	**1**	**0**	**0**	**2**	**0**	**0**	**2**	**0**	**2**	**17**
Greaves et al.[10]	**2020**	**2**	**2**	**2**	**1**	**2**	**2**	**2**	**1**	**0**	**0**	**2**	**1**	**2**	**2**	**2**	**2**	**25**
Liu et al.[11]	**2018**	**2**	**0**	**2**	**2**	**2**	**2**	**1**	**1**	**0**	**0**	**2**	**0**	**0**	**2**	**2**	**2**	**20**

1. *Scores for each item are between 0 and 2, where one point is awarded if the study had partial inclusion of methods that reduced bias and two points were awarded for full inclusion of methods to reduce risk of bias.
2. For full description of each individual item, access https://amstar.ca/Amstar_Checklist.php

References

1. Aceto P, Lai C, de Crescenzo F, et al. Cognitive decline after carotid endarterectomy: Systematic review and meta-analysis. European Journal of Anaesthesiology 2020;37:1066-74.
2. Cao L, Wang K, Gu T, Du B, Song J. Association between APOE epsilon 4 allele and postoperative cognitive dysfunction: A meta-analysis. International Journal of Neuroscience 2014;124:478-85.
3. Feinkohl I, Winterer G, Pischon T. Hypertension and risk of post-operative cognitive dysfunction (POCD): A systematic review and meta-analysis. Clinical Practice and Epidemiology in Mental Health 2017;13:27-42.
4. Feinkohl I, Winterer G, Pischon T. Obesity and post-operative cognitive dysfunction: a systematic review and meta-analysis. Diabetes/Metabolism Research and Reviews 2016;32:643-51.
5. Feinkohl I, Winterer G, Pischon T. Diabetes is associated with risk of postoperative cognitive dysfunction: A meta-analysis. Diabetes/Metabolism Research and Reviews 2017;33.
6. Feinkohl I, Winterer G, Pischon T. Associations of dyslipidaemia and lipid-lowering treatment with risk of postoperative cognitive dysfunction: a systematic review and meta-analysis. Journal of epidemiology and community health 2018;72:499-506.
7. Feinkohl I, Winterer G, Spies CD, Pischon T. Cognitive Reserve and the Risk of Postoperative Cognitive Dysfunction. Deutsches Arzteblatt international 2017;114:110-7.
8. Fu C, Lin J, Gong G, Zhong W, Chen H, Luo X. Inflammatory markers in postoperative cognitive dysfunction for patients undergoing total hip arthroplasty: a meta-analysis. Aging clinical and experimental research 2021:1-12.
9. Ghezzi ES, Ross TJ, Davis D, Psaltis PJ, Loetscher T, Keage HAD. Meta-Analysis of Prevalence and Risk Factors for Cognitive Decline and Improvement After Transcatheter Aortic Valve Implantation. The American journal of cardiology 2020;127:105-12.
10. Greaves D, Psaltis PJ, Davis DHJ, et al. Risk Factors for Delirium and Cognitive Decline Following Coronary Artery Bypass Grafting Surgery: A Systematic Review and Meta-Analysis. Journal of the American Heart Association 2020;9:e017275.
11. Liu X, Yu Y, Zhu S. Inflammatory markers in postoperativedelirium (POD) and cognitive dysfunction (POCD): A meta-analysis of observational studies. PLoS ONE 2018;13.
